# Morphological and Molecular Identification of *Physaloptera praeputialis* Infection and Description of Associated Gastric Lesions in the European Wildcat (*Felis silvestris*)

**DOI:** 10.3390/pathogens15090921

**Published:** 2026-09-01

**Authors:** Pablo Matas-Méndez, Alberto Benito-Peña, Lorena Esteban-Sánchez, Rocio Checa Herraiz, Francisco Ponce-Gordo, Marta Mateo-Barrientos

**Affiliations:** 1 Facultad de Veterinaria, Universidad Alfonso X el Sabio, Villanueva de la Cañada, 28691 Madrid, Spain; pmatamen@uax.es (P.M.-M.); abenipea@uax.es (A.B.-P.); 2 Departamento de Microbiología y Parasitología, Facultad de Farmacia, Universidad Complutense, 28040 Madrid, Spain; lorees01@ucm.es (L.E.-S.); rocichec@ucm.es (R.C.H.)

**Keywords:** felids, *Felis silvestris*, fibroplastic gastric nodules, *Physaloptera praeputialis*, Spain, spirurine nematodes

## Abstract

*Physaloptera praeputialis* is a common gastric spirurine of domestic and wild felids worldwide, but its pathological importance in European felids and the molecular characterisation of European isolates remain poorly documented. This study identifies *P. praeputialis* in European wildcats (*Felis silvestris*) from central Spain using morphological and molecular approaches and describes the associated gastric lesions. Thirty free-ranging wildcats were examined by necropsy, coproscopical analysis, and post-mortem inspection. Adult *Physaloptera* specimens were recovered from 11/30 (36.7%) wildcats, and they were identified morphologically and by 18S rRNA sequencing as *P. praeputialis*. Three infected wildcats presented well-demarcated intramural fibroplastic gastric nodules characterised by marked fibrosis, abundant collagen deposition, reactive fibroblasts, and eosinophil-rich inflammatory infiltrates surrounding intralesional adult female nematodes containing embryonated eggs. Molecular analysis of worms from the lesions confirmed their identity as *P. praeputialis*. Gross and histopathological features of these nodules were indistinguishable from those traditionally attributed to *Cylicospirura* spp. These findings provide the first direct evidence that *P. praeputialis* can induce fibroplastic gastric nodules in felids and should be considered in the differential diagnosis of lesions classically attributed to *Cylicospirura* spp. They also highlight the value of integrating morphological, molecular, and histopathological approaches in the diagnosis of gastric spirurine infections.

## 1. Introduction

Within the order Rhabditida, gastric nematodes of the genera *Physaloptera* (Physalopteridae) and *Cylicospirura* (Spirocercidae) are among the most frequently reported spirurines infecting domestic and wild felids worldwide [1,2,3,4,5,6,7]. Both genera have indirect life cycles involving arthropod intermediate hosts and a wide range of vertebrate paratenic hosts, facilitating trophic transmission to carnivores [8]. Adult worms inhabit the stomach of their definitive hosts, where they may induce chronic inflammatory lesions of variable severity [9,10,11,12].

Among felids, *Physaloptera praeputialis* is considered the most common species of the genus *Physaloptera* [8]. It has an almost cosmopolitan distribution in domestic cats [13,14,15,16,17] and has also been reported in several wild felids worldwide [2,6,18,19,20,21,22]. In Europe, *Physaloptera* spp. infections are rarely cited in domestic cats (*Felis catus*) [16] but they occur frequently in European wildcats (*Felis silvestris*), with prevalence values varying and potentially exceeding 50%, depending on the geographical area and the employed detection method [3,7,22,23,24,25,26]. Adult worms attach firmly to the gastric mucosa. Although infections are generally regarded as subclinical, they have occasionally been associated with chronic gastritis, erosions, ulceration, vomiting and weight loss in domestic cats [4,10]. To our knowledge, there is no data available on the pathology in wild felids.

In contrast, species of the genus *Cylicospirura* have been widely reported in wild felids from several continents [5,12,27,28,29], including Europe, where *Cylicospirura petrowi* and *Cylicospirura felineus* have been described in European wildcats [3,7,22,26]. Unlike *Physaloptera* spp., adult *Cylicospirura* spp. develop within the gastric wall and characteristically induce fibroplastic gastric nodules associated with chronic inflammation [1,11,12,27,30]. Consequently, gastric nodules in wild felids are generally considered suggestive of *Cylicospirura* infection.

In the Iberian Peninsula, there are no reports of *Physaloptera* in domestic cats, but it has previously been reported in European wildcats and Iberian lynxes (*Lynx pardinus*) [19,23,24], and adult specimens were morphologically identified as *P. praeputialis* [19,23]. However, no molecular confirmation of *P. praeputialis* has been reported in Spain. Moreover, the pathological importance of this parasite in free-ranging European (and Iberian) wild felids remains poorly understood. In previous studies based on opportunistic necropsies of animals found dead, adult parasites have been detected, but no associated nodules have been reported [3].

Therefore, the aims of the present study were to (i) identify the spirurine nematodes recovered from European wildcats using complementary morphological and molecular approaches, (ii) confirm their specific identity by sequencing the nuclear 18S rRNA gene, and (iii) characterise the gross and histopathological gastric lesions associated with the detected spirurine infection.

## 2. Materials and Methods

### 2.1. Study Population and Sample Collection

A total of 30 European wildcats (*F. silvestris*) found dead were included in this study. Carcasses were recovered by wildlife rehabilitation centres operating under the authority of the Regional Environmental Delegations of the Autonomous Communities of Castilla-La Mancha and of Castilla y León (Spain). Four wildcats originated from Castilla-La Mancha and 26 from Castilla y León. The study population comprised 24 males and 6 females, and all animals were adults (>1 year old). To preserve parasitic stages, carcasses were stored at −20 °C until necropsy. Animals and samples were collected under the corresponding permits issued by the Regional Environmental Delegations (Castilla-La Mancha: DGPFEN/SEN/avp_21_103_bis; Castilla y León: AB/is. Exp. AUES/CYL/001/2021).

### 2.2. Necropsy and Parasite Recovery

A complete necropsy was performed on each animal. The gastrointestinal tract, extending from the distal oesophagus to the rectum, was removed intact to prevent contamination of the abdominal cavity, and it was opened longitudinally. Particular attention was paid to the stomach, and the presence, location and gross appearance of gastric lesions were recorded.

Faecal samples collected from the distal colon or rectum were stored at 4 °C until coproscopical examination, while the contents of the other parts of the gastrointestinal tract were thoroughly washed and sieved to recover helminths. These were rinsed in phosphate-buffered saline (PBS) and preserved in 70% ethanol at 4 °C until morphological and molecular analyses.

The prevalence of spirurine nematode infection was calculated as the percentage of infected animals with respect to the total number of examined wildcats. Prevalence of the remaining gastrointestinal parasites detected by coproscopical examination or necropsy was calculated using the same approach.

### 2.3. Faecal Examination

Faecal examination was performed as a complementary diagnostic method to compare coproscopical findings with parasites recovered during necropsy. Faecal samples were processed using a modified Telemann sedimentation technique. Briefly, approximately 1–3 g of faeces was homogenised in 5% acetic acid (ThermoFisher Scientific, Waltham, MA, USA), filtered through a double layer of cotton gauze, and adjusted to approximately 5 mL of filtrate. An equal volume of diethyl ether (Merck KG&A, Darmstadt, Germany) was then added, and the mixture was gently homogenised and centrifuged at 1500 rpm for 3 min using a Mixtasel BL centrifuge (JP Selecta, Barcelona, Spain). The resulting sediment was examined microscopically as unstained wet mounts under 400–1000× magnification following the method originally described by Telemann [31] and modified by Allen and Ridley [32].

### 2.4. Morphological Identification of Spirurine Parasites

Adult nematodes recovered from the stomach were clarified in lactophenol and identified morphologically according to the keys of Yamaguti [33], Soulsby [9] and Gibbons [34]. Spirurine identification was based on the morphology of the cephalic and cuticular structures, such as the caudal papillae and posterior cuticular sheath structures. Representative specimens were subsequently selected for molecular confirmation.

### 2.5. Histopathological Examination

All macroscopically visible gastric nodules observed during necropsy were collected for histopathological evaluation. Tissue samples were fixed in 4% buffered formaldehyde (methanol-stabilised, pH 7.0; PanReac AppliChem, Barcelona, Spain) for at least 72 h at room temperature, routinely processed, embedded in paraffin wax (Merck), sectioned at 4 μm using a rotary microtome (Leica RM2125 RTS, Leica Microsystems, Wetzlar, Germany), and stained with haematoxylin and eosin (H&E) (Papanicolau Harris haematoxylin, Bio-Optica Milan S.p.A., Milan, Italy; Eosin Y solution, alcoholic, Merck).

Selected sections were additionally stained with Masson’s trichrome (Merck) to assess collagen deposition (fibrosis), following the method described by Cruz et al. [35]. Histopathological changes and parasitic structures were described by light microscopy. Because only transverse histological sections were available within the gastric nodules, species identification based on morphology alone was not considered reliable. Therefore, parasite identification was based in genetic analysis.

### 2.6. Molecular Analyses

Several spirurine specimens from each individual wildcat were selected for individual molecular identification. Prior to DNA extraction, each nematode was rinsed several times in PBS to remove residual ethanol. Each individual was sectioned into small fragments and digested overnight at 70 °C under continuous agitation in 200 μL of TE buffer (100 mM Tris-HCl, Merck; 1 mM EDTA, Merck; pH 8.0), 200 μL of 10% sodium dodecyl sulphate (SDS; Merck) and 15 μL of Proteinase K (1 μg/μL) (ThermoFisher Scientific). Genomic DNA was subsequently extracted using the phenol–chloroform protocol described by Sambrook and Russell [36]. DNA was recovered in 100 μL of Milli-Q water and stored at −20 °C until further analysis.

To confirm the identity of intralesional nematodes, DNA was also extracted from formalin-fixed paraffin-embedded (FFPE) gastric nodules. Briefly, 10 µm paraffin sections were deparaffinized by two consecutive xylene washes and rehydrated through a graded ethanol series to distilled water. DNA was subsequently extracted from the entire tissue section using the same phenol–chloroform protocol as described for adult nematodes.

For the genetic identification of the spirurines, a partial fragment of the nuclear small subunit ribosomal RNA (18S rRNA) gene was PCR-amplified using the forward primer Physa-F (5′-GCGAACGGCTCATTATAA) and the reverse primer Physa-R (5′-AATTTCACCTCTCAGCA) [37]. Amplification reactions were performed in a final volume of 25 μL using PuReTaq Ready-To-Go PCR Beads (Merck). Each reaction contained 5 μL of template DNA and 2 μL of each primer solution.

Amplifications were carried out in a Mastercycler Gradient thermal cycler (Eppendorf, Hamburg, Germany) following the protocol described by Maldonado et al. [37], with minor modifications: initial denaturation at 94 °C for 3 min, followed by 32 cycles of denaturation at 94 °C for 45 s, annealing at 47 °C for 45 s, and extension at 72 °C for 1 min, with a final extension at 72 °C for 1 min.

PCR products were separated by electrophoresis on 1% agarose gel stained with Pronasafe (Condalab, Torrejón de Ardoz, Spain) and visualised under ultraviolet light using a NuGenius UV transilluminator (Syngene, Cambridge, UK). Amplicons showing the expected size were purified using the QIAquick PCR Purification Kit (QIAGEN, Hilden, Germany) according to the manufacturer’s instructions.

Purified PCR products were sequenced in both directions at the Genomics Unit of the Universidad Complutense de Madrid using the corresponding amplification primers. Sequencing reactions were performed on an ABI Prism 3730XL Genetic Analyzer (Applied Biosystems, Foster City, CA, USA).

Chromatograms were edited and assembled using ChromasPro v2.1.10 (Technelysium Pty Ltd., South Brisbane, Australia). Consensus sequences were compared with those available in the GenBank/EMBL/DDBJ databases using the Basic Local Alignment Search Tool (BLAST) available through the National Center for Biotechnology Information (NCBI) (https://blast.ncbi.nlm.nih.gov/Blast.cgi; accessed: 27 July 2026) to confirm species identity.

## 3. Results

### 3.1. Gastrointestinal Parasites

Overall, 28/30 (93.3%) wildcats harboured at least one gastrointestinal parasite, and mixed infections involving two or more parasite taxa were detected in 13/30 (43.3%) animals. The gastrointestinal parasites detected by coproscopical examination and necropsy are summarised in Table 1.

**Figure 1:**
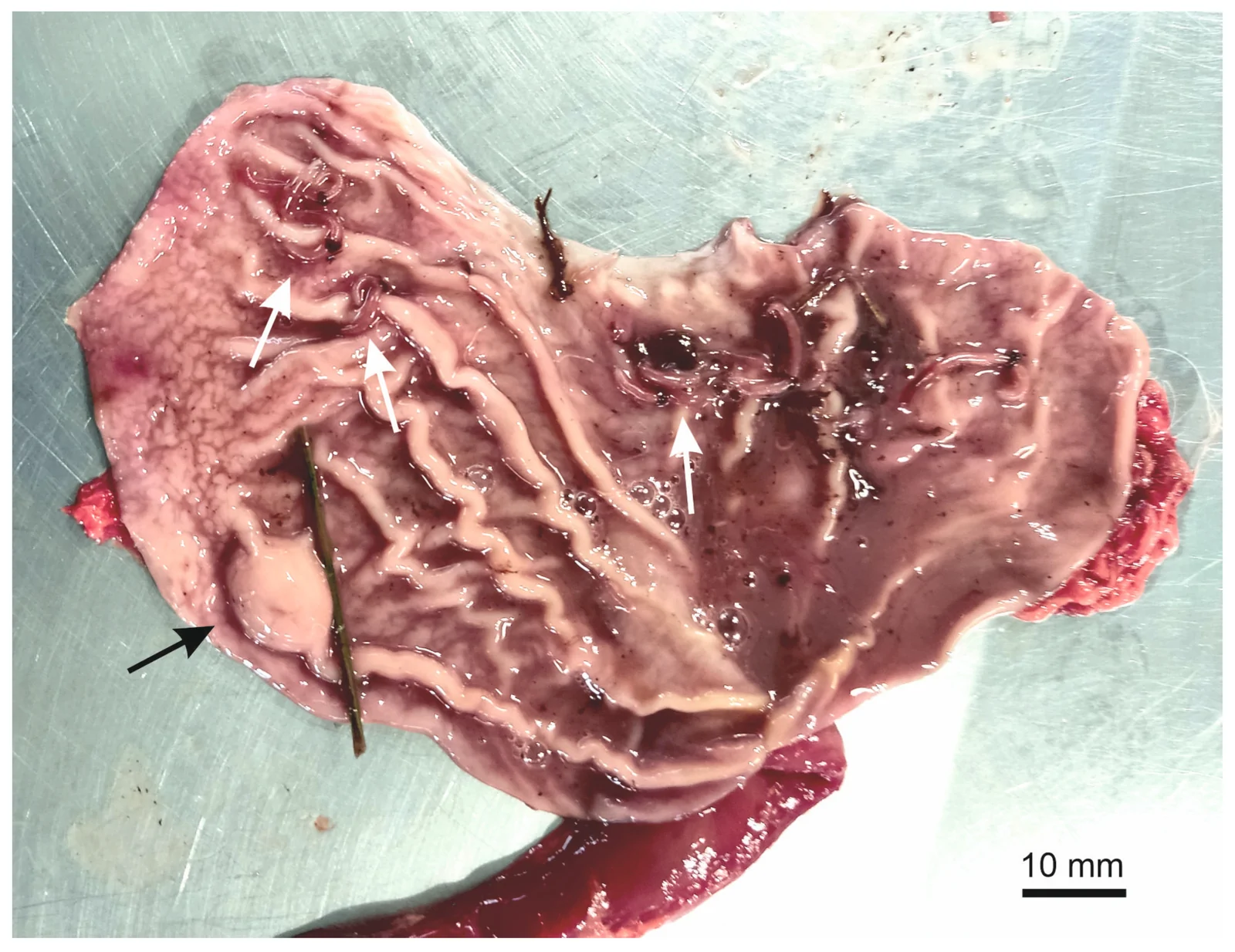
Necropsy of a stomach of a wildcat showing adult spirurines compatible with *Physaloptera praeputialis* attached to the mucosa (white arrows) and a gastric nodular lesion (black arrow).

Among the helminths recovered at necropsy, spirurines compatible with *Physaloptera* spp. were found attached exclusively to the gastric mucosa of 11/30 (36.7%) wildcats (Figure 1), whereas *Toxocara cati* was recovered from the small intestine of 19/30 (63.3%) animals.

### 3.2. Morphological and Molecular Identification of Spirurines Recovered from the Gastrointestinal Tract

All adult nematodes recovered from the stomach displayed morphological features consistent with *P. praeputialis*. No adult specimens of *Cylicospirura* or other gastric spirurines were recovered from any of the examined wildcats.

**Figure 2:**
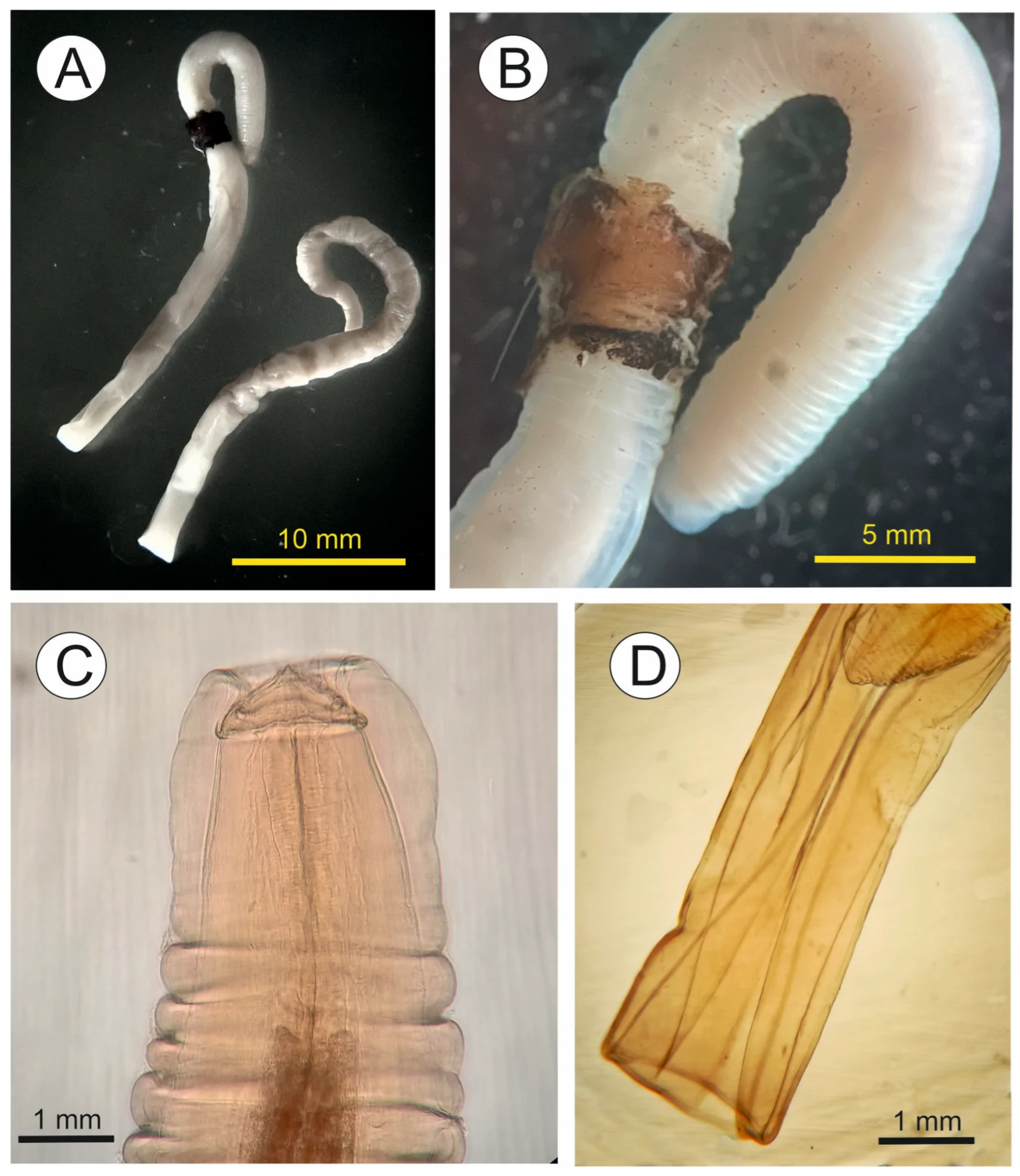
Adult specimens of *Physaloptera praeputialis* recovered from the stomach of a wildcat. (**A**) Female and male adults. (**B**) The female shows the characteristic brown cement ring surrounding the vulva. (**C**) Detail of the triangular pseudolabia. (**D**) Detail of the cuticular sheath at the posterior extremity.

The spirurine nematodes all exhibited the characteristic morphological features of the genus *Physaloptera*, including two large triangular pseudolabia armed with internal teeth, and in males, four pairs of pedunculated caudal papillae together with sessile papillae surrounding the cloaca. Both sexes presented a distinct caudal cuticular sheath, whereas females exhibited the characteristic brown cuticular ring surrounding the vulva, consistent with the diagnosis of *P. praeputialis* (Figure 2).

Morphological identification was fully supported by molecular analyses. The 18S rRNA sequence obtained in this study was submitted to GenBank under the accession number PZ721969 and showed 100% nucleotide identity with *P. praeputialis* recovered from a European wildcat in Romania (GenBank accession PZ016044) and 98.87% with an isolate from a stray domestic cat in India (MW410927).

### 3.3. Gastric Lesions Associated with Spirurine Infection

Fibroplastic gastric nodules were observed in 3/11 (27.3%) wildcats infected with *P. praeputialis*. No gross gastric lesions were observed in the remaining infected animals. The number of *P. praeputialis* recovered ranged from 6 to 87 nematodes per stomach. No apparent association was observed between parasite burden and the presence of gastric nodules, as some wildcats without nodules harboured higher parasite burdens than nodule-positive animals. In all three animals, numerous adult nematodes were attached to the gastric mucosa; none were macroscopically observed associated with the nodules; however, additional adults were identified within the fibroplastic nodule in the histological analysis. Grossly, the lesions consisted of well-circumscribed, firm, rounded intramural nodules measuring approximately 1 cm in diameter (Figure 1).

**Figure 3:**
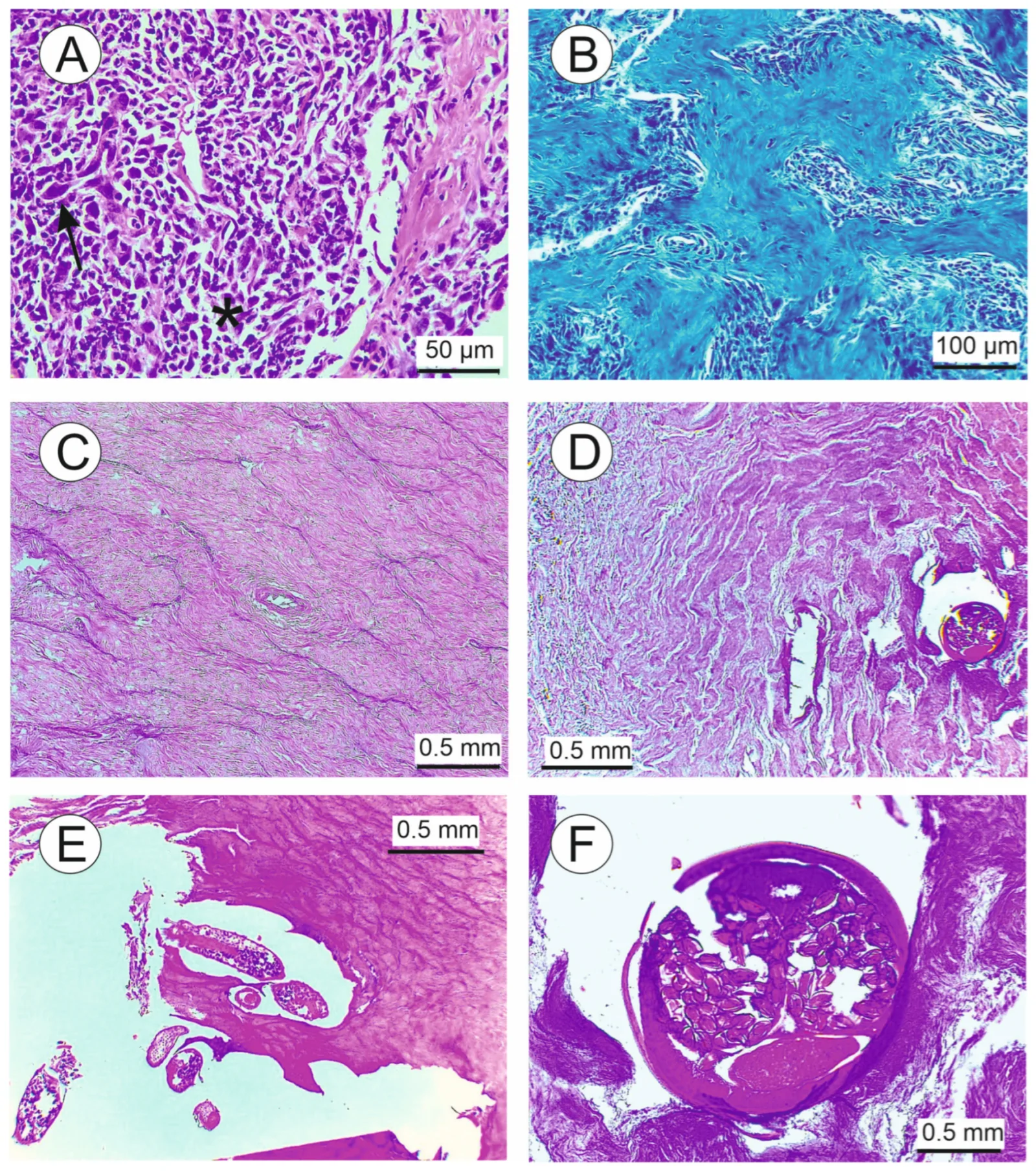
(**A**) Case 1. Reactive fibroblasts (arrow) and mixed inflammatory infiltrate, predominantly composed of eosinophils (asterisk), within the gastric lesion. H&E stain, ×40. (**B**) Case 1. Dense collagen bundles highlighted by Masson’s trichrome stain within the gastric nodule. Masson’s trichrome, ×20. (**C**) Case 2. Dense collagen bundles within the gastric nodule. H&E stain, ×10. (**D**) Case 3. Moderate fibrosis surrounding a parasitic section within the gastric nodule. H&E stain, ×10. (**E**) Case 2. Multiple cross-sections of adult nematodes located in the centre of the gastric lesion. H&E stain, ×10. (**F**) Case 3. Higher magnification of a transverse section of an adult nematode, showing the cuticle, intestine and paired uteri containing embryonated eggs. H&E stain, ×20.

Histologically, all nodules were centred within the gastric submucosa and were characterised by marked fibrosis with abundant collagen deposition, as confirmed by Masson’s trichrome staining. The lesions were histopathologically classified according to the grading criteria proposed for FGESF, considering the depth of involvement of the gastrointestinal wall, the degree of fibrosis, the proliferation of reactive fibroblasts, the presence of necrosis and the intensity of the eosinophilic infiltrate [38]. Lesion severity varied among cases. Cases 1 (Figure 3A,B) and 2 (Figure 3C) exhibited extensive, densely packed collagen bundles arranged concentrically around the lesion centre (grade III, severe), whereas collagen deposition was comparatively less pronounced in case 3 (Figure 3D), in which the connective tissue appeared more loosely organised (grade II, moderate).

A mixed inflammatory infiltrate was evident, particularly in case 1, and consisted predominantly of eosinophils together with smaller numbers of neutrophils, lymphocytes and macrophages. Reactive fibroblasts were abundant within the fibrotic tissue, especially in the most cellular lesion (Figure 3A,B).

The central portion of each nodule contained one or more cross-sections of adult nematodes surrounded by concentric fibrosis. Parasite sections displayed preserved morphological structures, including the cuticle, intestinal tract and paired uteri containing embryonated eggs, consistent with adult female *P. praeputialis* (Figure 3E,F). No identifiable male nematode anatomical features were observed in the examined transverse sections; however, the absence of male nematodes cannot be definitively established because the sections examined may not have captured male individuals.

Molecular analysis of DNA extracted from paraffin-embedded nodules confirmed that the intralesional parasites corresponded to *P. praeputialis*; the resulting 18S rRNA sequence was identical to that obtained from the adult nematodes.

## 4. Discussion

The overall prevalence of gastrointestinal parasites observed in European wildcats (93.3%) is consistent with previous studies conducted on European wild felids, confirming that endoparasitic infections are a common in this species [3,39,40]. Mixed infections involving several taxa of helminths and protozoa were also common, which likely reflects the opportunistic feeding behaviour of wildcats and their exposure to multiple food-borne parasites [41]. Although these findings provide additional information on the gastrointestinal parasite community of European wildcats, the present study focuses specifically on spirurines and their associated gastric lesions. None of the other parasites detected are known to induce fibroplastic gastric nodules.

The present study provides the first combined morphological, molecular and pathological characterisation of *P. praeputialis* infecting European wildcats in Spain. Although *Physaloptera* spp. have previously been reported in European wildcats and Iberian lynx, these records were based exclusively on coproscopical findings [24] or morphological examination of adult worms [19,23]. To the best of our knowledge, this study therefore represents the first molecular confirmation of *P. praeputialis* in Spain and one of the few genetically characterised isolates currently available from European felids [16].

The prevalence of *P. praeputialis* observed in this study (36.7%) falls within the range previously reported for European wildcats, in which infection rates vary from approximately 12% to over 50%, depending on the geographical region and the diagnostic method employed [3,22,23,24,25,26]. As reported for other carnivores, these differences probably reflect regional variation in the abundance of intermediate and paratenic hosts together with differences in prey composition [26]. Because European wildcats feed predominantly on small mammals, but also consume reptiles, birds and amphibians, trophic transmission of *Physaloptera* is likely to occur frequently [8,41].

In live animals, *Physaloptera* spp. infection is generally investigated by faecal examination. However, the eggs cannot be reliably identified to the species level by routine coproscopical examination, and the sensitivity of this method may be limited. These limitations should be considered when comparing prevalence estimates based on coproscopical examination with those obtained from necropsy-based studies.

Morphological examination of the recovered nematodes revealed the diagnostic characteristics of *P. praeputialis*, particularly the distinctive brown cuticular ring surrounding the female vulva, together with the characteristic cephalic and caudal structures. Although these morphological features allow reliable species identification, molecular characterisation was performed as a complementary approach to confirm the diagnosis and provide the first genetic evidence of this species in Spain. The 18S rRNA sequence was identical to that recently reported from domestic cats and European wildcats in Romania [16], suggesting that genetically highly similar or identical populations of *P. praeputialis* circulate among geographically distant European felid populations. Given the limited molecular information currently available for this species, further studies from different geographical regions will be necessary to clarify its genetic diversity, geographical distribution and host range.

One of the most important findings of this study was the demonstration that fibroplastic gastric nodules contained adult *P. praeputialis*. To the best of our knowledge, this is the first direct evidence linking this species to fibroplastic gastric nodules in felids. Gastric nodules in felids have traditionally been attributed to *Cylicospirura* spp., which has been reported in several felid species, including the domestic cat [1,30] and the European wildcat [3,5,7,11,12,27,29]. Importantly, no adult *Cylicospirura* or other gastric spirurines were recovered from any of the examined wildcats. Together with the molecular identification of the intralesional nematodes, this finding confirms *P. praeputialis* as the only aetiological agent of the fibroplastic gastric nodules described in the present study. Characterisation of the lesions associated with *P. praeputialis* infection represents one of the few available approaches to assess its potential impact on the health of felid populations.

Grossly, the nodules closely resembled those described in *Cylicospirura* infections, appearing as well-demarcated intramural gastric thickenings. Histologically, they were characterised by marked fibrosis with abundant collagen deposition and eosinophil-rich inflammatory infiltrates, features that have also been reported in gastric lesions associated with *Cylicospirura* spp. [11,42]. No consistent or specific microscopic features were identified that would allow reliable differentiation between *P. praeputialis*-associated and *Cylicospirura*-associated fibroplastic nodules. In particular, the type and distribution of fibrosis and the inflammatory infiltrate did not provide a reliable basis for distinguishing between the two infections. This marked gross and histopathological similarity indicates that nodules produced by both genera may be undistinguishable in the absence of complete adult specimens; in such cases, genetic analysis should be required. Consequently, *P. praeputialis* should be included in the differential diagnosis of fibroplastic gastric nodules in wild felids, particularly in Europe, where both genera have been reported in European wildcats.

Histological examination revealed intralesional nematodes displaying the characteristic morphology of adult female spirurines, including paired uteri containing numerous embryonated eggs. Although adult *Physaloptera* are generally described as parasites firmly attached to the gastric mucosa [8,9], the present findings indicate that some individuals may become deeply embedded within chronic fibroplastic lesions of the gastric wall. This deep embedding within the fibroplastic tissue may explain why the nematodes were not macroscopically identified within the gastric nodules. These observations also highlight the value of integrating histopathological and molecular approaches to accurately establish the aetiology of gastric nodules in wild felids.

Only three of the eleven wildcats infected with *P. praeputialis* developed fibroplastic gastric nodules. However, parasite burden alone does not appear to explain the development of these lesions, as some animals with high parasite loads did not develop nodules. The chronicity of infection could not be determined because the duration of infection was unknown, although the presence of dense collagen bundles and eosinophilic inflammation within the nodules is compatible with a chronic fibroproliferative response. Similarly, immunological parameters were not assessed, so it is not possible to determine whether individual differences in the host response contributed to the development of the lesions. Thus, differences in the duration of infection and in individual host responses may contribute to the observed variability in lesion presentation, although this possibility should be investigated in future studies.

The histopathological features observed in this study also showed notable similarities to feline gastrointestinal eosinophilic sclerosing fibroplasia (FGESF), a chronic inflammatory condition of domestic cats characterised by abundant collagen deposition, proliferation of reactive fibroblasts and eosinophilic inflammation [43,44,45]. The dense concentric collagen bundles surrounding the parasites, together with the predominance of eosinophils in the best-preserved lesions, support the hypothesis that prolonged parasite persistence may act as a plausible inflammatory trigger for a chronic fibroproliferative response resembling that described in FGESF. Although FSEGF is primarily characterised in the domestic cat (*Felis catus*), histologically similar gastrointestinal lesions have been reported in other felids, particularly in pumas (*Puma concolor*) [46].

Interestingly, lesion severity differed among the three affected wildcats. Whereas one case showed abundant eosinophilic inflammation together with marked fibroblast proliferation, the remaining lesions in the other two wildcats were dominated by dense mature collagen with comparatively little inflammatory infiltrate. Similar variation has been described in FGESF and has been interpreted as reflecting different stages of lesion evolution, in which an initial eosinophil-rich inflammatory response progressively evolves towards mature fibrosis [44,45,46,47]. Although the small number of cases in the present study precludes drawing conclusions regarding lesion progression, the similar pattern observed here is compatible with a chronic fibroproliferative response.

The limited inflammatory infiltrate observed in two of the lesions may also have been influenced by post-mortem autolysis. Because all wildcats included in this study were found dead, most following road traffic collisions, varying degrees of autolysis were unavoidable at necropsy. Eosinophils are particularly susceptible to post-mortem degradation, which may lead to underestimation of the original inflammatory response [48,49]. This interpretation is supported by the fact that the best-preserved case also displayed the most prominent eosinophilic infiltrate.

Eosinophils are recognised mediators of fibrosis through the production of profibrotic cytokines, particularly transforming growth factor-β, which promotes fibroblast activation and collagen deposition. In domestic cats, the aetiology of FGESF often remains uncertain and multiple chronic antigenic stimuli, including bacteria, fungi, dietary hypersensitivity and parasites, have been proposed [38,45,50]. The demonstration of adult *P. praeputialis* embedded within the fibroplastic nodules provides strong evidence of an association between the parasite and the lesions observed in the present cases. However, this finding does not establish that these lesions represent classical FGESF as described in domestic cats. Rather, the lesions described here support the hypothesis that chronic gastric spirurine infection may act as a plausible inflammatory trigger for a fibroproliferative response resembling FGESF.

## 5. Conclusions

This study provides the first molecular confirmation of *P. praeputialis* infecting European wildcats in Spain and provides compelling evidence that *P. praeputialis* can be associated with fibroplastic gastric nodules in felids. These findings expand current knowledge of the pathology associated with *Physaloptera* infections and show that fibroplastic gastric nodules should not be considered pathognomonic of *Cylicospirura* infection in felids. The results further highlight the importance of integrating morphological, molecular and histopathological approaches for the diagnosis of gastric spirurine infections. Additional studies involving larger numbers of animals will be required to determine the frequency, pathogenesis and clinical importance of these lesions in domestic, feral and wild felid populations.

## Figures and Tables

**Table 1:** Frequency of gastrointestinal parasites detected by coproscopical examination and necropsy in European wildcats (*Felis silvestris*) from central Spain. Data are expressed as number of positive animals (*n*) and prevalence (%). Percentages were calculated using the total number of examined wildcats (*N* = 30). Dash (-) indicates that the parasites (protists) were not investigated at necropsy.

Parasite	Coproscopy *n* (%)	Helminth Recovered at Necropsy *n* (%)
Spirurine eggs	8 (26.6)	11 (36.6)
*Toxocara cati*	17 (56.6)	19 (63.3)
*Trichuris* spp.	3 (10.0)	0 (0.0)
*Capillaria* spp.	3 (10.0)	0 (0.0)
*Hydatigera taeniaeformis* sensu lato	0 (0.0)	19 (63.3)
*Dipylidium caninum*	0 (0.0)	1 (3.3)
*Joyeuxiella* spp.	0 (0.0)	3 (10.0)
*Cystoisospora* spp.	14 (46.6)	-
*Toxoplasma gondii*	1 (3.3)	-

## Data Availability

All new data is presented in the article or available in public databases (GenBank accession number PZ016044).
